# Radiation and anti-PD-L1 synergize by stimulating a stem-like T cell population in the tumor-draining lymph node

**DOI:** 10.21203/rs.3.rs-3921977/v1

**Published:** 2024-03-06

**Authors:** Yang Shen, Erin Connolly, Meili Aiello, Chengjing Zhou, Prasanthi Chappa, Haorui Song, Patan Tippitak, Tarralyn Clark, Maria Cardenas, Nataliya Prokhnevska, Annapaola Mariniello, Meghana S. Pagadala, Vishal R. Dhere, Sarwish Rafiq, Aparna H. Kesarwala, Alexandre Orthwein, Susan N. Thomas, Mohammad K. Khan, J. Brandon Dixon, Gregory B. Lesinski, Michael C. Lowe, Haydn Kissick, David S. Yu, Chrystal M. Paulos, Nicole C. Schmitt, Zachary S. Buchwald

**Affiliations:** 1 Department of Radiation Oncology and Winship Cancer Institute, Emory University, Atlanta, GA, USA; 2 Bioinformatics Graduate Program, Georgia Institute of Technology, Atlanta, GA, USA; 3 Department of Urology and Winship Cancer Institute, Emory University, Atlanta, GA, USA; 4 Marc and Jennifer Lipschultz Precision Immunology Institute, Icahn School of Medicine at Mount Sinai (ICMMS), New York City, NY, USA; 5 Department of Pathology and Immunology, University of Geneva, Geneva, Switzerland; 6 Department of Microbiology and Immunology, Emory University School of Medicine, Atlanta, GA, USA; 7 Medical Scientist Training Program, University of California San Diego, La Jolla, CA USA; 8 George W. Woodruff School of Mechanical Engineering, Georgia Institute of Technology, Atlanta, GA, USA; 9 Parker H. Petit Institute for Bioengineering and Bioscience, Georgia Institute of Technology, Atlanta, GA, USA; 10 Wallace H. Coulter Department of Biomedical Engineering, Georgia Institute of Technology and Emory University, Atlanta, GA, USA; 11 Department of Hematology and Medical Oncology and Winship Cancer Institute, Emory University, Atlanta, GA, USA; 12 Department of Surgery and Winship Cancer Institute of Emory University, Atlanta, GA, USA; 13 Department of Urology and Winship Cancer Institute, Emory University, Atlanta, GA, USA; 14 Department of Otolaryngology - Head and Neck Surgery and Winship Cancer Institute, Emory University, Atlanta, GA, USA; 15 These authors contributed equally; 16 Lead contact

**Keywords:** Radiation, immunotherapy, PD-1, PD-L1, checkpoint blockade, stem-like T cells, Tcf-1, tumor-draining lymph node, abscopal effect

## Abstract

Radiotherapy (RT) and anti-PD-L1 synergize to enhance local and distant (abscopal) tumor control. However, clinical results in humans have been variable. With the goal of improving clinical outcomes, we investigated the underlying synergistic mechanism focusing on a CD8+ PD-1+ Tcf-1+ stem-like T cell subset in the tumor-draining lymph node (TdLN). Using murine melanoma models, we found that RT + anti-PD-L1 induces a novel differentiation program in the TdLN stem-like population which leads to their expansion and differentiation into effector cells within the tumor. Our data indicate that optimal synergy between RT + anti-PD-L1 is dependent on the TdLN stem-like T cell population as either blockade of TdLN egress or specific stem-like T cell depletion reduced tumor control. Together, these data demonstrate a multistep stimulation of stem-like T cells following combination therapy which is initiated in the TdLN and completed in the tumor.

## INTRODUCTION

CD8+ T cells play a critical role in the anti-tumor immune response. However, chronic antigen exposure in cancer leads to CD8+ T cell exhaustion with upregulation of markers including PD-1, Tim3 as well as epigenetic changes.^1–3^ Blockade of PD-1 promotes CD8+ T cell expansion and reinvigoration leading to robust clinical responses in many different types of cancer.^4–7^ Interestingly, CD8+ PD-1+ T cells within the tumor microenvironment are heterogenous with subsets including progenitor stem-like CD8+ T cell and terminally differentiated effector like cells (TE).^8,9^ The stem-like subset, in contrast to the TE, expresses the transcription factor Tcf-1. Following PD-1/L1 blockade, the stem-like CD8+ T cell subset expands and differentiates into the TE subset with granzyme B expression and the capacity for tumor cell killing.^10,11^ Unfortunately, anti-PD-1 monotherapy achieves durable tumor control in only a subset of patients highlighting the need to develop novel combinatorial approaches that improve outcomes.

Radiotherapy (RT) is effective as a local treatment and is known to also have immunomodulatory effects. On occasion, tumor regression outside the radiation field occurs via immune-stimulation, a process known as the abscopal effect.^12–14^ RT mediates this effect, in part, by acting as in-situ vaccine while broadening the T cell receptor repertoire and recruiting naïve/antigen experienced T cells to the anti-tumor immune response. Importantly, this effect can synergize with single agent checkpoint blockade.^15–18^ Combined RT and PD-1/L1 blockade has recently shown encouraging clinical potential^18,19^, however, there remains an urgent need to more deeply understand the underlying mechanism to improve response rates, in particular the impact RT has on T cell exhaustion and reinvigoration.

The tumor-draining lymph node (TdLN) is important for a robust RT or anti-PD-1/L1 stimulated immune response.^20–24^ More recent studies have shown that the TdLN acts as a reservoir for stem-like T cells.^22,25,26^ This population of stem-like T cells in the TdLN serve as developmental precursors for the intra-tumoral population, and they continuously migrate from the TdLN to the tumor under basal conditions.^25^ Once in the tumor they undergo further differentiation into TE. Additionally, Huang *et al.* demonstrated that the TdLN stem-like population is an important mediator of the anti-PD-1/L1 response.^22^ Finally, our earlier work suggested this TdLN reservoir may also be important for the RT alone stimulated immune response.^21^ Together, these findings suggest that the TdLN stem-like T cell population is important for the observed synergy between RT and anti-PD-L1.

Here, using murine models of melanoma, we address the direct effect of RT or combination therapy on the TdLN stem-like T cells. We find that combination therapy enhances the differentiation of this subset in the TdLN via a novel transcriptional program followed by substantial expansion and continued differentiation into TE in the tumor. The resulting improvement in both local and distant tumor control depends both on this TdLN subset and its egress from the lymph node.

## RESULTS

### Combination RT + anti-PD-L1 promotes an increase in intra-tumoral stem-like and terminal effector CD8+ T cells

**Figure 1.**
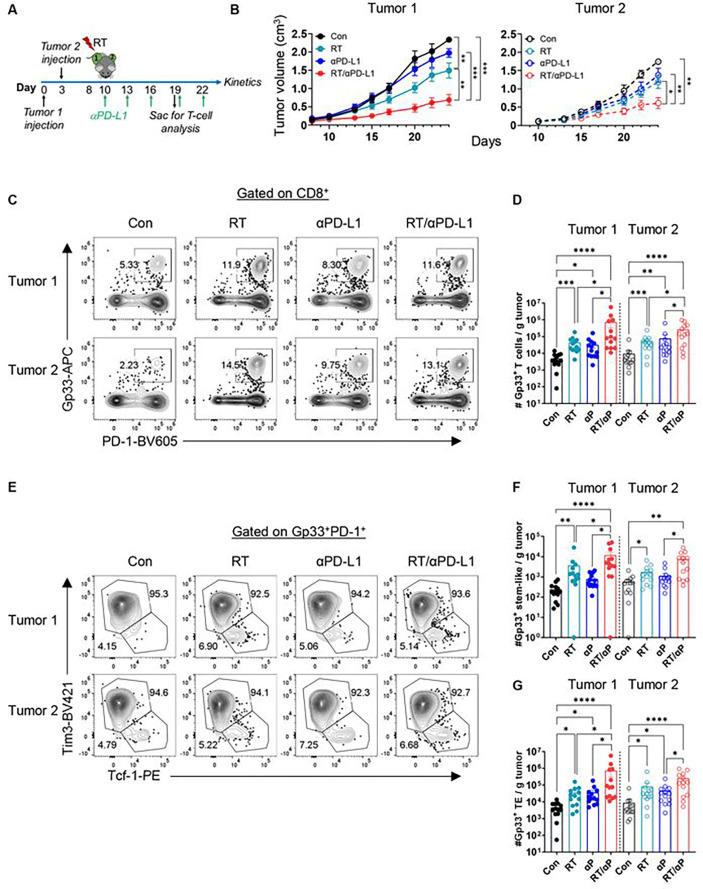
RT + anti-PD-L1 promote an increase in intra-tumoral stem-like and terminal effector CD8+ T cells. **(A)** Experimental schema. **(B)** Tumor growth kinetics for the RT targeted tumor I and the distant (abseopal tumor) tumor 2 demonstrating enhanced control with combination therapy. Data reflect 2 separate experiments combined (n=10 total). **(C)** Representative plots of Gp.33+ PD-1+ T cells gated on CD8 in tumor 1 and tumor 2 under different treatment conditions. **(D)** Quantitation plots for number of Gp33+ T cells per gram tumor. **(E)** Representative plots of Tim3− Tcf-1+ stem-like and Tim3+ Tcf-1− TE gated on CD8+ PD-1+ Gp33+ T cells. **(F)** Quantitation plots for number of stem-like T cells per gram tumor. **(G)** Quantitation plots for number of TE T cells per gram tumor. *:p<0.05, **:p<0.01, ***:p<0.001, ****:p<0.0001 by ANOVA. Data reflect 3 separate experiments combined (n = 13 total).

We previously showed that RT alone can enhance the anti-tumor immune response leading to improved tumor control in a CD8+ T cell dependent manner.^21^ Here, to interrogate the impact of combination RT and anti-PD-L1 on local and abscopal tumor control as well as CD8+ T cell subsets, B16F10 cells expressing the lymphocytic choriomeningitis (LCMV) glycoprotein (B16F10GP), which allow for the identification of tumor-specific T cells^21^, were sequentially implanted on bilateral flanks of wt C57BL/6 mice (Figure 1A). Sequential implantation of the flank tumors was done to model metachronous metastatic disease. Tumor 1, the initially injected tumor, was treated with 10 Gy × 1 fraction of RT (**Figure S1A**) with or without anti-PD-L1 starting day 10 post-implantation.^21^ Mice were sacrificed 9 days after treatment initiation (day 19), for tissue analysis (Figure 1A). Tumor 1 and tumor 2 growth were significantly reduced with RT alone (Figure 1B, **S1B-S1C**). In contrast, anti-PD-L1 alone had minimal effect on the growth of tumor 1 or tumor 2 consistent with the known resistance of this cell line to PD-1 based therapy.^27,28^ Combination RT and anti-PD-L1, however, dramatically slowed the growth of both the irradiated tumor 1 and the unirradiated tumor 2 to a greater extent than either monotherapy (Figure 1B, **S1B-S1C**). We performed the same kinetic analysis in both the parental B16F10 cell line and another melanoma cell line (YUMM1.7) and found similar robust synergy between RT + anti-PD-L1 at both the primary and abscopal site (**Figure S1D**).

Next, we investigated the anti-tumor immune response and found the number of bulk CD8+ T cells in tumor 1 and tumor 2 were not significantly increased with RT or anti-PD-L1 alone while combination therapy demonstrated significant increases in both tumors (**Figure S1E**). We then evaluated tumor specific CD8+ Gp33+ T cells and again found a significant increase in tumor 1 and tumor 2 following combination treatment compared to untreated or monotherapy (Figure 1C–1D). Importantly, within the tumor-specific CD8+ Gp33+ T cell population, both the stem-like and TE subsets also increased in tumor 1 and tumor 2 following RT + anti-PD-L1 with no significant changes in their relative frequencies (Figure 1E–1G, **S1F-G**). To complete this initial analysis, CD8+ T cell function following combination therapy was assessed by restimulating bulk tumor CD8+ T cells ex-vivo with Gp33 peptide. We found that RT or anti-PD-L1 monotherapy did not significantly increase the frequency nor the number of IFN-γ+ and IFN-γ+ TNF-α+ T cells. However, combination therapy led to significant increases in both groups (**Figures S1H-S1K**).

### The TdLN supplies the tumor with stem-like CD8+ T cells following RT + anti-PD-L1

**Figure 2.**
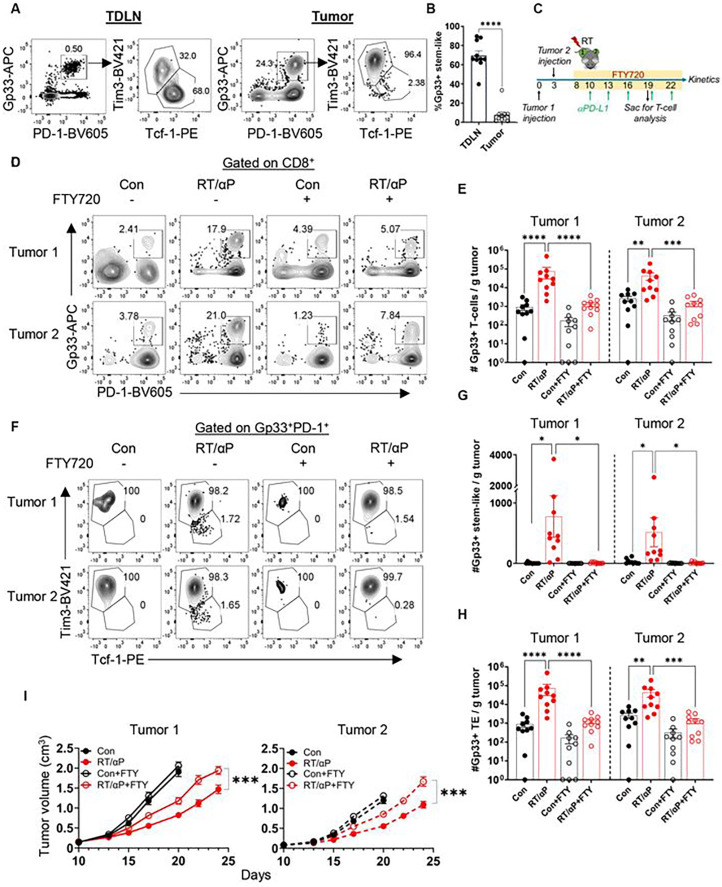
The TdLN supplies the tumor with stem-like CD8+ T cells following RT + anti-PD-L1. **(A)** Representative plots of PD-1 + Gp33+ T cells and stem-like vs. TE in the tumor and TdLN. **(B)** Quantitation of the stein-like T cell frequency in the TdLN and tumor. **(C)** Experimental schema with yellow bar representing FTY720 administration in drinking water. **(D)** Representative plots gated on CD8+ of PD-1+ Gp33+ T cells in the tumor under different treatment conditions with or without FTY720. **(E)** Quantitation of the number of Gp33+ T cells per gram tumor as in D. **(F)** Representative plots gated on antigen specific subsets under different treatment conditions with or without FTY720. **(G)** Quantitation of the number of antigen specific stem-like T cells per gram tumor as in F. **(H)** Quantitation of the number of antigen specific TE T cells per gram tumor as in F. Combined data from 2 separate experiments (n=10 total per group). **(I)** Tumor kinetics under different treatment conditions with and without FTY720. Combined data from 2 experiments (n=15 total). *:p<0.05, **:p<0.01. ***:p<0.001 two-tailed t test.

Prior studies have demonstrated the importance of stem-like T cells in the anti-PD-1/L1 stimulated response to chronic viral infections and cancer.^10,11^ Additionally, our group and others have shown that the tumor-draining lymph node (TdLN) is an important reservoir for stem-like T cells which supply the tumor.^21,22,25,26^ Tumor antigen specific cells are primarily found in the TdLN rather than the non-TdLN or other secondary lymphoid organs like the spleen (**Figure S2A**). We have previously shown that disrupting this reservoir via TdLN immunodepletion, impaired RT alone mediated immunostimulation.^21^ These data suggest that this stem-like T cell reservoir in the TdLN may be important for the synergy between RT and anti-PD-L1. To evaluate this hypothesis, we again confirmed that the majority of the Gp33+ T cells in the TdLN were stem-like cells (Tim3-Tcf-1+) while most of the Gp33+ T cells in the tumor were TE (Figure 2A–2B). Mice were then treated with FTY720 prior to RT or anti-PD-L1 to prevent lymphocyte egress from the TdLN and other secondary lymphoid organs (Figure 2C). The percentage of circulating total lymphocytes, CD4+, and CD8+ T cells in the blood decreased significantly upon FTY720 administration (**Figures S2B-S2D**). In both tumors, administration of FTY720 blocked the increase of total CD8+ and Gp33+ T cells observed following combination therapy. (Figure 2D–2E, **S2E-S2G**). Examination of the subsets found the increased numbers of tumor-antigen specific stem-like and TE in the tumor induced by combination therapy was also abolished by FTY720 treatment (Figures 2F–2H, **S2H-S2J**). Notably, FTY720 also attenuated the slowing of tumor growth by combination RT + anti-PD-L1 (Figure 2I, **S2K**).

In the TdLN, the frequency and number of total CD8+ T cells following RT + anti-PD-L1 remained unchanged with or without FTY720 (**Figure S3A-S3B**). In contrast, the frequency of CD8+ PD-1+ Gp33+ T cells significantly increased with FTY720 treatment, and the total number of PD-1+ Gp33+ T cells approached significance (**Figure S3C-S3E**). Importantly, the number of Gp33+ stem-like T cells was significantly increased in the TdLN with FTY720 and combination therapy (**Figures S3F-S3H**); the TE did not reach significance (**Figure S3I-S3J**). Together, these results support the hypothesis that the increase in tumor stem-like T cells following RT + anti-PD-L1 depends on their egress from the TdLN.

### ScRNA-seq analysis identified multiple CD8+ PD-1+ Tcf-1+ T cell subsets in the TdLN

**Figure 3.**
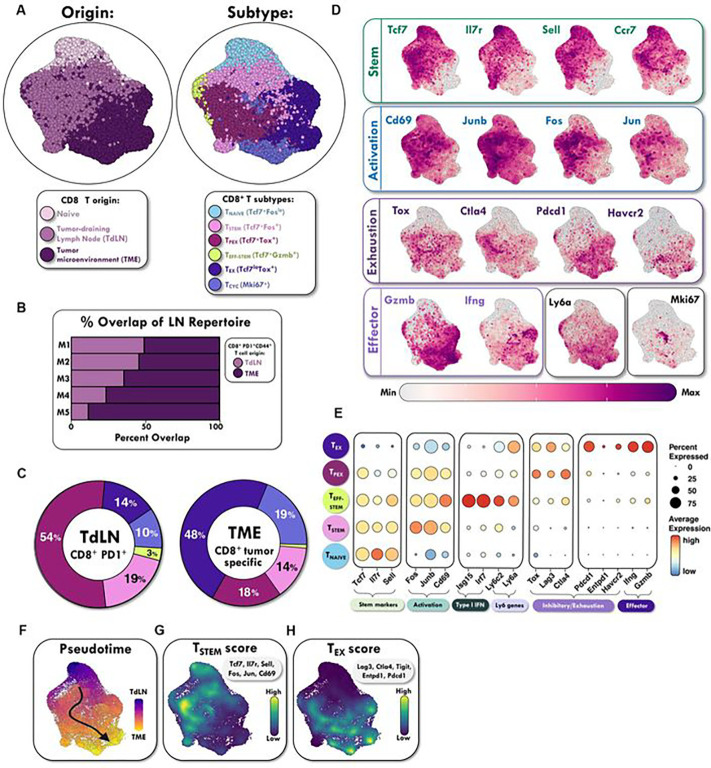
ScRNA-scq analysis identified multiple CD8+ FD-1+ Tcf-1+ T cell subsets in the TdLN. **(A)** UMAP identified six major cell populations in the TdLN and tumor microenvironment (TME). **(B)** TCR sequencing demonstrates substantial overlap between antigen experienced polyclonal CD8+ T cell populations in the tumor and TdLN. **(C)** Quantitation of different subset frequencies in the TdLN and the TME; legend same as in panel (A). **(D)** Feature plots showing expression level and location of relevant markers. **(E)** Average expression and percent expressing various markers in the different T cell subsets. **(F)** Pseudotime analysis evaluating the developmental trajectory of stem-like to effector T cells from the TdLN to the TME. **(G)** T_STEM_ found in early pseudotime, while **(H)** T_EX_ found in late pseudotime. N=5 mice.

To further interrogate the impact of combination therapy on the stem-like T cells in the TdLN, we performed single cell RNA-seq (scRNA-seq) first under untreated conditions on sorted TdLN CD8+ PD-1+ T cells. Naïve cells were sequenced separately and introduced as controls as were published tumor infiltrating lymphocytes (TIL) data from similar tumors models.^29^ Unsupervised clustering and visualization of cell types with uniform manifold approximation and projection (UMAP) identified six major cell populations in the TdLN and tumor (Figure 3A). After batch correction, both visual and quantitative evaluations in cell identity and transcriptional signatures showed very similar population clustering to recently published datasets of adoptively transferred tumor specific T cells found in the TdLN (**Figure S4A**).^22^ To confirm that the TdLN-derived CD8+ PD-1+ T cells in our dataset were clonally related to the CD8+ T cells within the tumor, we performed bulk-TCR sequencing of sorted CD8+ PD-1+ T cells from paired TdLN and tumor mice samples. Despite being a polyclonal pool, up to 50% of the TdLN CD8+ PD-1+ repertoire overlap with the tumor suggesting that a large fraction of antigen experienced TdLN T cells are tumor specific even under untreated conditions (Figure 3B). Next, clustering and visualization of the scRNA-seq CD8+ T cell clusters revealed the presence of both previously described CD8+ T cell subtypes and new groups, including three CD8+ PD-1+ T_STEM_ populations in the TdLN: *(i)* a T_STEM_ subset with *Tcf7* and *Fos* expression; *(ii)* a T_PEX_ subset with *Tcf7* and *Tox* expression *(iii)* and a non-canonical T_EFF-STEM_ subset with both *Tcf7* and *Gzmb* expression (Figure 3D–3E). As expected, CD8+ cells in the tumor were featured by the emergence of exhausted Tox-positive *Tcf7−*negative CD8+ T cells (T_EX_) accounting for about 50% of total CD8+ PD-1+ T cells in the tumor, whereas *Tcf7−*positive T_STEM_ subsets showed a strong distribution preference in the TdLN, with the *Tcf7+ Tox+* T_PEX_ population being the most abundant (Figure 3C).

A hallmark of T cells exhaustion is persistent expression of markers including *Tox, Ctla4, Entpd1, Pdcd1, Havcr2*, and others, many of which were expressed in the tumor T_EX_ subset (Figure 3D–3E). Some of these markers of exhaustion (*Lag3, Ctla4, Tox*) appeared in the T_PEX_ subset (Figure 3E), distinguishing the T_PEX_ cells from other stem-like subsets. Tumor-enriched CD8+ T_EX_ cells also expressed high levels of *Gzmb* and *Ifng* (Figure 3D–3E), genes associated with CD8+ effector T cell function. By contrast, TdLN-enriched CD8+ T cells projected mostly onto markers associated with stemness (*Tcf7, Il7r, Sell, Ccr7*) and activation (*Jun, Fos, Cd69, Junb*) (Figure 3D). Of note, we observed that this small population of T_EFF-STEM_ cells were defined by the co-expression of an interferon-stimulated gene signature and the murine *Ly6* gene complex namely *Ly6a* and the memory marker *Ly6c* (Figure 3E).^30^

We then assessed how spatial location (TdLN vs. tumor) impacts CD8+ T cell differentiation. To visualize how CD8+ T cell differentiation is regulated from the node to the TME, we constructed single-cell trajectories. All CD8+ T cells in the TdLN and tumor were placed on these trajectories based on changes in their transcriptomes. Consistent with the clustering analyses, T_STEM_ cells from the TdLN were predominantly distributed throughout the early pseudotime, whereas T_EX_ cells were found mostly in later pseudotime, showing a strong trajectory pattern originating from the TdLN to the tumor (Figure 3F). To reveal the overall pattern of T cell phenotypes across the TdLN and tumor, we identified two highly correlated modules consisting of genes associated with a stemness module in the TdLN and an exhaustion module in the tumor (Figures 3G–3H) consistent with prior reports.^22,25^ These data confirm that T_STEM_ and T_EX_ sit at opposite ends of a T cell differentiation spectrum.

### Combination RT + anti-PD-L1 induces a Tcf-1+ effector-stem subset in the TdLN

**Figure 4.**
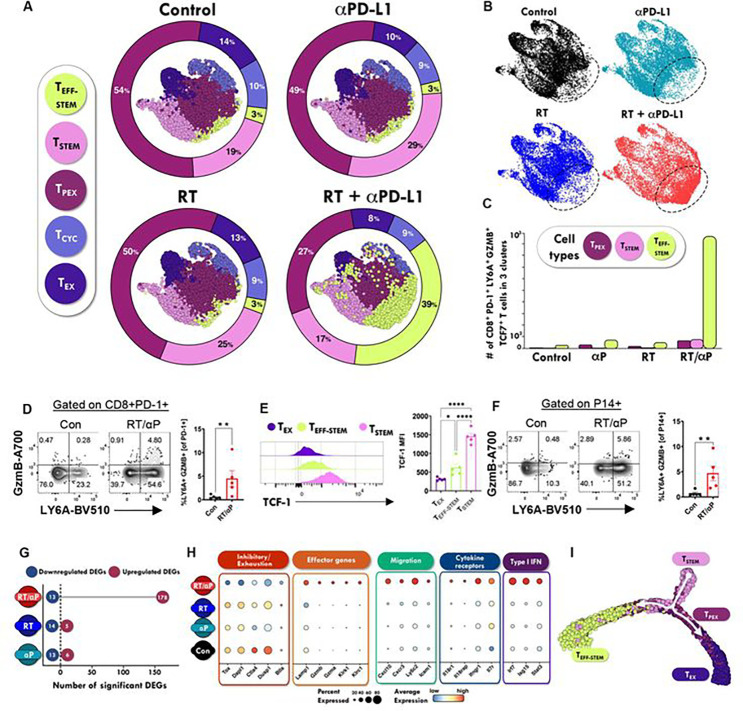
Combination RT + anti-FD-L1 induces a Tcf-1+ effector-stem subset in the TdLN. **A) UMAP** and quantitation demonstrating the major cell CD8+ PD-1+ T cell populations in the I’dLN under different treatment conditions. **(B)** UMAP by treatment condition with novel population present in RT + anti-PD-L1 group circled. **(C)** Quantitation of number of Ly6a+ GzmB+ Tcf7+ T cells found in each cluster broken down by treatment condition. **(D)** Representative flow plot and quantitation demonstrating the presence of Ly6a+ GzmB+ T cells with combination therapy. **(E)** Ly6a+ GzmB+ cells have an intermediate Tcf-1 expression level. **(F)** A fraction of adoptively transferred PI4 T cells specific for Gp33 also adopt a stem-effector phenotype with combination therapy. **(G)** Differentially expressed genes relative to untreated controls. **(H)** Select gene clusters were differentially expressed in the combination therapy group. **(I)** Pseudotime analysis suggest a distinct developmental pathway for T_EFF-STEM_ compared to traditional stem-like to effector development. *:p<0.05. **:p<0.01, ****:p<0.0001 by two-tailed t test. N=5 mice per group.

Next, to evaluate the impact of combination therapy on the TdLN T cell population, we again performed scRNA-seq on sorted CD8+ PD-1+ T cells 7 days after treatment with anti-PD-L1, RT alone or combination therapy. We sequenced 38,578 cells with an average of 1,928 cells per sample (n=5 mice per treatment group). Annotation of unsupervised clusters revealed 5 major populations of CD8+ T cells. Interestingly, scRNA-seq analysis revealed that the gene expression profile of CD8+ T cells was similar in untreated mice compared to mice treated with anti-PD-L1 or RT alone. However, dimensionality reduction indicated substantial phenotypic differences between mice treated with RT + anti-PD-L1 combination therapy compared with those receiving monotherapy. Specifically, a >10-fold expansion of the CD8+ T_EFF-STEM_ population was observed in the TdLNs of mice treated with RT + anti-PD-L1 (Figure 4A–4B, **S4B**). Additionally, decreased frequencies of T_PEX_ and T_STEM_ populations were observed in the TdLN of mice treated with combination therapy compared to mice treated with anti-PD-L1 or RT alone (Figure 4A–4B). No significant differences were observed in other cell populations.

We then assessed the numbers of stem-like CD8+ PD1+ T cells that co-express *Tcf7, Ly6a*, and the effector molecule *Gzmb*. T_STEM_ and T_PEX_ subsets represent the two most abundant TdLN CD8+ T cell populations in mice treated with anti-PD-L1 or RT alone, and they comprised 98% of the *Tcf7* expressing cells which do not express *Gzmb* and *Ly6a*. By contrast, more than 10^3^ CD8+ PD-1+ T cells in the T_EFF-STEM_ population co-expressed *Tcf7, Ly6a*, and *Gzmb* after combination therapy compared to <100 such cells in mice that were untreated or given anti-PD-L1 or RT alone (Figure 4C). To determine whether this T_EFF-STEM_ was also detectable by flow cytometry using these 3 markers (Tcf-1, Ly6A, GzmB), we evaluated TdLN CD8+ PD-1+ T cells under untreated or following combination therapy and found a significant increase in the Ly6A+ GzmB+ population (Figure 4D). This Ly6a+ GzmB+ population also expressed significantly more Tcf-1 compared to tumor infiltrating T_EX_, and significantly less than the canonical T_STEM_ in the TdLN (Figure 4E) consistent with the transcriptional results. To confirm this population was also present in a tumor-antigen specific T cell population, we adoptively transferred 2.5e6 P14 T cells (specific for Gp33) and treated the mice with combination therapy on Day 10 post tumor inoculation. In the TdLN P14 T cell population, we were again able to detect a significant increase in the Ly6A+ GzmB+ population with combination therapy (Figure 4F).

To gain further insights into therapy-induced changes in gene expression, we calculated the number of differentially expressed genes (DEG) for each treatment group relative to untreated controls. Concurrent therapy with both RT and anti–PD-L1 led to a greater number of differentially upregulated CD8+ PD-1+ T cell DEG compared with either agent alone (Combo = 178 genes; RT = 5 genes; anti–PD-L1 = 6 genes), with little overlap between genes upregulated across the three treatment cohorts (Figure 4G, **S4C, Supplementary table 2**). Relatively few genes were significantly downregulated in all three treatment cohorts relative to untreated controls (Combo = 13 genes; anti–PD-L1 = 14 genes; RT = 13 genes) (Figure 4G, **Supplementary table 2**). Notable pathway enrichment analysis further highlighted the gene expression changes in CD8+ T cells induced by combination therapy. Specifically, expression of inhibitory/exhaustion molecules (*Tox, Ctla4, Dusp1, Dapl1* and *Btla*) were downregulated in CD8+ PD-1+ T cells from mice TdLN treated with combination therapy compared with CD8+ T cells isolated from untreated mice or after anti-PD-L1 or RT therapy alone (Figure 4H, **S4D**). In contrast to decreased RNA levels for genes relating to exhaustion, there was significant enrichment of genes associated with effector molecules (*Gzmb, Gzma, Klrk1, Lamp1,* and *Tbx21*), cytokine receptors (*Il18r1, Il18rap, Ifngr1,* and *Il7r*), and type I IFN (*Irf7, Isg15, Stat3*) following combination therapy. One of the most significant alterations in CD8+ T cells after combination therapy was the upregulation of genes associated with migration (*Cxcl10, Cxcr3, Ly6c2*, and *Icam1*), which may indicate enhanced migratory capacity of TdLN CD8+ T cells following combination therapy (Figure 4H, **S4D**). Taken together, these results show that combination therapy reprograms CD8+ PD-1+ T cells to display effector cell properties with increased cytotoxic and migratory capacity, while maintaining a stem-like phenotype. Additionally, scRNA-seq analysis uncovered two potentially distinct developmental paths within the stem-like CD8+ PD1+ T cell population of the TdLN, suggesting a potential divergence towards distinct fates or functions following combination therapy (Figure 4I).

### RT promotes the expansion and differentiation of TdLN stem-like T cells which is enhanced with anti-PD-L1

**Figure 5.**
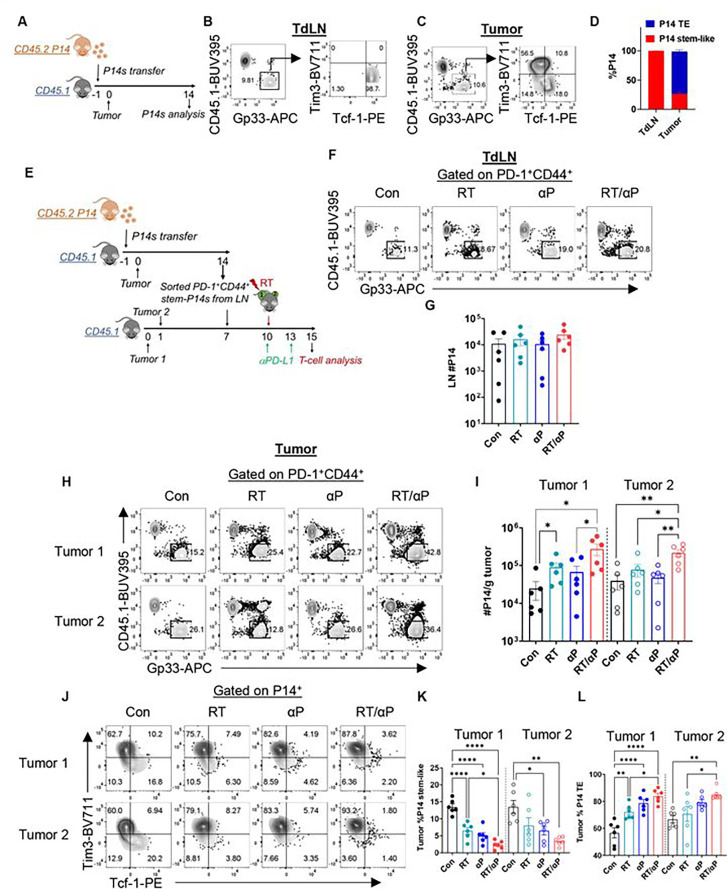
RT promotes the expansion and differentiation of TdLN stem-like T cells which is enhanced with anti-PD-L1. **(A)** Experimental schema. **(B)** Representative flow plots showing the P14 DTR+ T cells differentiating into stems in the TdLN and **(C)** effectors in the tumor. **(D)** Frequency of stems and TE in the TdLN vs tumor. **(E)** Experimental schema with serial adoptive transfer. **(F)** Representative flow plot of gating on transferred P14s in the TdLN under different treatment conditions. **(G)** Quantitation of the number of P14s in the TdLN by treatment condition. **(H)** Representative flow plot of gating on transferred P14s in the tumor under different treatment conditions. **(I)** Quantitation of P14s per gram tumor. **(J)** Representative flow plots of P14 T cell subsets in the tumors. **(K)** Frequency of stem-like and **(L)** TE in the tumors. *:p<0.05, **:p<0.01. ****:p<0.0001 by ANOVA. Data reflect combined data from two separate experiments (n=6 total).

Prior data has shown that anti-PD-L1 monotherapy promotes the expansion and differentiation of stem-like T cells.^11^ Our scRNA-seq analysis suggests that combination therapy also promotes stem-like T cell differentiation while inducing a unique transcriptional program in the TdLN. To further interrogate these observations and determine the impact of RT alone and combination therapy on stem-like T cell differentiation, we performed a serial adoptive transfer experiment using P14 T cells. First, we sacrificed mice 14 days after tumor injection to confirm that naïve P14 T cells would migrate to the TdLN and the tumor and adopt a stem-like and TE phenotype following initial adoptive transfer (Figure 5A). P14 T cells were recovered in both the TdLN and tumor, and the vast majority of the cells (99%) were stem-like in the TdLN while displaying a TE phenotype in the tumor, alike endogenous cells (Figure 5B–D). Next, CD44+ PD-1+ Tim3− stem-P14s were sorted from the TdLNs and transferred into B16F10GP tumor-bearing mice, followed by treatment with RT with or without anti-PD-L1 3 days later (Figure 5E, **S5A**). We did not find any significant difference in the number of P14s or P14 Tcf-1+ T cells in the TdLN with either monotherapy or combination (Figure 5F–5G, **S5B**). We did, however, find phenotypic changes in the TdLN population with combination therapy. In line with our transcriptional analysis, we observed a significant increase in the number of P14+ Ly6A+, GzmB+ Tcf-1+ T cells in the TdLN with RT + anti-PD-L1 but not monotherapy (**Figure S5C-S5D**). These results confirm that this novel subset is derived from the TdLN stem-like precursor.

We then turned our attention to the tumors and found that there was a significant increase in the number of P14 T cells in tumor 1 for RT alone (Figure 5H–5I). The frequency of stem-like cells significantly decreased with RT alone with a concomitant increase in the frequency of TE cells demonstrating that RT alone can promote both expansion and differentiation of TdLN stem-like T cells in the targeted tumor (Figure 5J–5L). Importantly, combination therapy led to greater expansion of stem-like P14s in both tumor 1 and tumor 2 than monotherapy with enhanced differentiation of stem-like T cells into TE compared to RT alone (Figure 5H–5L). In the tumor, in contrast to the TdLN, there was a robust population of Ly6A+ GzmB+ cells in all groups including controls (**Figure S5E**). However, in all groups including combination therapy they were Tcf-1− (**Figure S5E-S5G**). Together, these data suggest that combination therapy initiates a novel differentiation program in the TdLN stem-like T cell population with subsequent migration, expansion and effector differentiation in the tumor.

### Stem-like CD8+ T cells are required for optimal synergy between RT and anti-PD-L1

**Figure 6.**
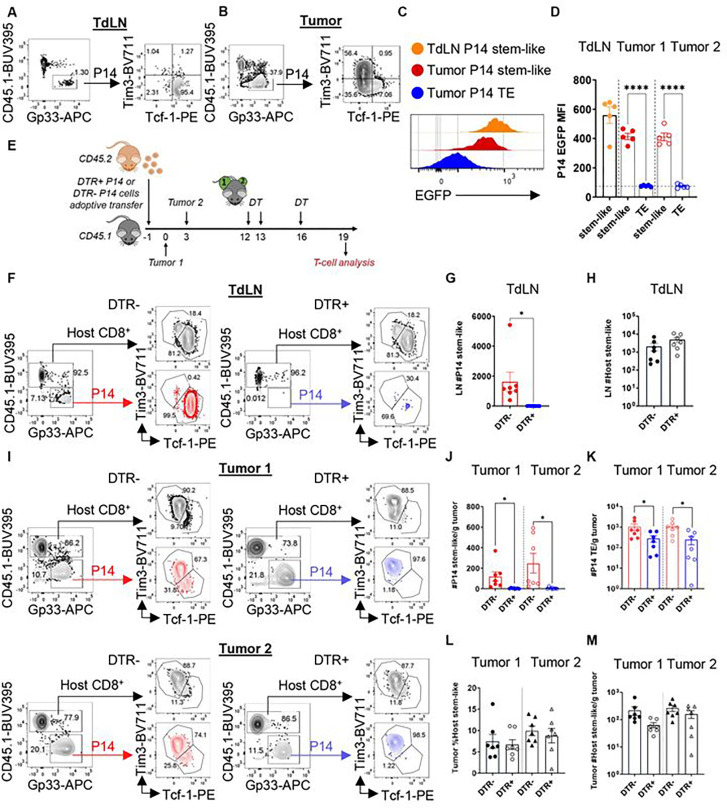
P14 Tcf7^DTR-eGFP^ allows for the specific depletion of stem-like T cells in the TdLN and tumor. **(A)** Representative (low plots showing the P14 DTR+ T cells differentiating into stems in the TdLN and **(B)** TE in the tumor. **(C)** Representative flow plots of eGFP MIT in different subsets. **(D)** eGFP MFI quantified by different subsets. **(E)** Experimental schema with diphtheria toxin (DT) administration. **(F)** Representative flow plots with gating on the endogenous and transferred P14 T cells in either the DIR− (red) or DTR+ (blue) recipients. **(G)** Quantitation of DIR− or DTR+ P14 stem-like T cells following DT administration. **(H)** Quantitation of endogenous stem-like T cells. **(I)** Representative flow plots with gating evaluating transferred and endogenous cells in the tumors. **(J)** Quantitation of PI4 stem-like and **(K)** TE per gram tumor. **(L)** Quantitation of endogenous stem-like and **(M)** TE per gram tumor. *:p<0.05. ****:p<0.0001 by two-tailed t test. Combined data from 2 separate experiments (n=7 total per group).

Given the substantial intra-tumoral expansion of TdLN derived stem-like T cells and differentiation into cytotoxic TE following combination therapy, we finally wished to determine whether this subset was required for the optimal tumor control induced by RT + anti-PD-L1. Thus, we generated a knock-in mouse which allowed for specific depletion of Tcf-1+ T cells. We inserted a diphtheria toxin receptor (DTR) P2A eGFP gene into the 3’ untranslated region of the Tcf7 locus using CRISPR technology (**Figure S6A**). CD45.2 Tcf7^DTR-eGFP^ were then bred with P14 mice to generate CD45.2 P14 Tcf7^DTR-eGFP^ (**Figure S6A**). To verify that P14 Tcf7^DTR-eGFP^ (P14 DTR+) differentiate into stem-like T cells expressing both Tcf-1 and eGFP, we adoptively transferred CD45.2 P14 DTR+ cells into CD45.1 mice one day before tumor injections. B16F10GP cells were then injected on bilateral flanks on day 0. We found P14 DTR+ T cells in both the tumor and TdLN (**Figure S6B**), and eGFP was highly expressed in the PD-1+ Tcf-1+ stem-like subset in both the tumor and TdLN, but not in the Tcf-1− Tim3+ T cells from the tumor on day 19 (Figure 6A–6D). Of note, stem-like T cell P14 DTR+ numbers in the TdLN and tumor were similar to P14 DTR− suggesting no differences in response to chronic antigenic stimulation (**Figure S6C-S6D**). Tumor growth kinetics for both tumor 1 and tumor 2 in P14 DTR+ versus DTR− recipients were indistinguishable (**Figure S6E**).

Next, we tested whether DT specifically depleted the Tcf-1+ T cell population. DTR− or DTR+ P14 were adoptively transferred followed by tumor inoculation and DT administration (Figure 6E). In both TdLN and the tumor, DT ablated PD-1+ Tcf-1+ cells from adoptively transferred P14 DTR+ but not from P14 DTR− littermate controls (Figure 6F–6G, 6I–6J). There was also a reduction in TE population (Figure 6K) attributable to the elimination of the precursor stem-like T cell. Importantly, endogenous stem-like T cells and TE were unchanged in either DTR− or DTR+ recipients (Figure 6H, 6L, 6M).

**Figure 7.**
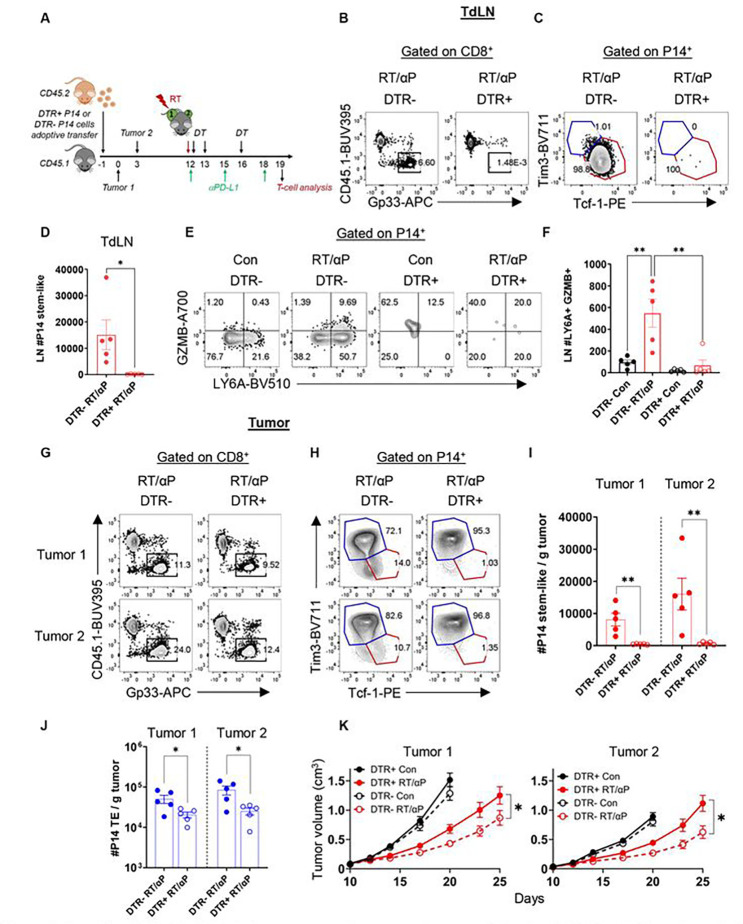
Stem-like CD8+ T cell depletion attenuates the synergy between RT and anti-PD-L1. **(A)** Experimental schema. **(B)** Representative flow plots of DTR− or DTR+ P14 cells and **(C)** subsets in the TdLN. **(D)** Quantitation of P14 stem-like T cells in the TdLN. **(E)** Representative flow plots of Ly6a+ GzmB+ cells in the TdLN. **(F)** Quantitation of Ly6a+ GzmB+ cells in the TdLN. **(G)** Representative flow plots of DTR− or DTR+ P14 cells and **(H)** subsets in the tumors. **(I)** Quantitation of the number of PI4 stem-like T cells and **(J)** TE per gram tumor. **(K)** Tumor growth kinetics under different treatment conditions with DTR+ or DTR− P14 cell transfer. *:p<0.05, **:p<0.01 by two-tailed t test. Data shown from a representative experiment n=5 per group, repeated 3 times.

Having validated specific depletion of stem-like T cells in CD45.1 recipient mice, we explored the impact of stem-like T cell depletion on RT + anti-PD-L1. To do this, we again adoptively transferred P14 DTR+ or P14 DTR− from littermate controls into CD45.1 and implanted tumors on bilateral flanks (Figure 7A) followed by combination therapy starting on Day 12. In the TdLN, the adoptively transferred stem-like T cell population was depleted only in P14 DTR+ recipients (Figure 7B–7D, **S6F**). Additionally, in the P14 DTR− recipient mice treated with RT + anti-PD-L1, we could detect the T_EFF-STEM_ (Ly6a+ GzmB+), but this subset was also completely depleted from the TdLN in the P14 DTR+ recipients (Figure 7E–7F). Next, we evaluated the tumor and found transferred T cells in both the P14 DTR+ and P14 DTR− recipients (Figure 7G, **S6G**). The stem-like T cell subset was again only depleted in P14 DTR+ recipients (Figure 7H–7I **S6H-I**). A reduction was also observed in the tumor TE subset again confirming that the stem-like T cells are necessary for TE production (Figure 7J, **S6J**). Finally, we evaluated tumor growth and found that with specific stem-like cells depletion, both local tumor control and the abscopal effect induced by combination RT and anti-PD-L1 were significantly reduced (Figure 7K, **S6K**). Tumor control was not completely abrogated as these mice still possess their endogenous stem-like and TE T cells. These results confirm that for optimal synergy between RT and anti-PD-L1 the TdLN stem-like T cell subset is required.

## DISCUSSION

The aim of our study was to explore the mechanism underlying the known synergy between RT and anti-PD-L1 focusing on a Tcf-1+ stem-like T cell subset. Here, using murine melanoma models, we found that combination therapy stimulates a novel differentiation program in TdLN stem-like T cells. Once in the tumor, these cells expand robustly and differentiate into terminal effectors. Finally, we showed that the optimal synergy for tumor control with RT and anti-PD-L1 depends on this TdLN subset. Based on these observations, we propose a multistep/multi-tissue expansion and differentiation model initiated in the TdLN and completed in the tumor. This model has several implications ranging from a fundamental biological standpoint to a clinical perspective.

Biologically, the finding that combined RT + anti-PD-L1 induces a novel differentiation program with more robust stem-like T cell proliferation is provocative. RT has been previously shown to promote the release of both cryptic/sequestered tumor antigen, IFN-I signaling, and damage associated molecular patterns (DAMPs) leading to enhanced APC maturation and T cell activation.^31,32^ Importantly, prior studies primarily focused on the intra-tumoral T cells to the exclusion of secondary lymphoid organ subsets.^17^ Our findings suggest that the RT induced antigen bolus and/or cytokine production is enough, by itself, to promote stem-like T cell expansion and differentiation initiated in the TdLN. This is further enhanced and modified by the presence of anti-PD-L1. This observation is highly novel as, to this point, robust stem-like T cell differentiation was thought to be primarily dependent on PD-1/L1 blockade. Whether APC migration from the tumor to the TdLN or whether antigen passively drains to the node following RT is an area of active investigation and the focus of future studies.

An additional intriguing finding was the T_EFF-STEM_ phenotype in the TdLN. T_EFF-STEM_, which were present at very small numbers at baseline, dramatically increased with combination therapy. An elegant recent paper from Hashimoto et al. demonstrated that following combined IL-2 + anti-PD-L1 treatment in chronic LCMV, a similar T cell phenotype expressing both Tcf7 and GzmB was induced.^33^ In their study, they found distinct epigenetic signatures in virus specific T cells following IL-2 or IL-2 + anti-PD-L1 treatment compared to anti-PD-L1 alone suggesting this T cell subset is not simply a transitory or intermediate phenotype between the stem-like and TE. In our case, we were able to identify this T_EFF-STEM_ subset only in the TdLN. This suggests that RT + anti-PD-L1 may be promoting a novel differentiating intermediate which migrates to the tumor where it robustly expands and completes the differentiation program. Future studies will more fully elucidate these findings.

Clinically, there are also broad and far-reaching implications from this work. A number of clinical trials evaluating combination RT + checkpoint blockade have had mixed to underwhelming results.^18,19,34,35^ Notably, many clinical trials have focused on treating larger volumes with elective nodal irradiation, in particular head and neck cancer.^35^ More recent data has confirmed that elective nodal irradiation or surgical nodal disruption can blunt both the local and distant radio-immunotherapy stimulated anti-tumor response.^20,21,36,37^ Our results show, definitively, why that is the case. They also suggest that a neoadjuvant approach to combination therapy, especially for melanoma, where the draining nodes are not disturbed by either surgery or radiation will have the potential for greater synergy and anti-tumor immune responses. Additionally, metastatic sites of disease targeted for induction of an abscopal response must have robust nodal drainage to effectively stimulate an immune response.

Finally, future studies will investigate methods to overcome the dependency on the TdLN. As noted, many clinical scenarios have tumors which either lack robust nodal drainage or it is difficult to assess. Therefore, if a TdLN like microenvironment can be replicated within the tumor or other secondary lymphoid organs, then this anatomical and immunologic limitation may be overcome.

### Limitations of this study

In this study, we evaluated the importance of the TdLN and stem-like T cells for the synergy of RT + anti-PD-L1 in murine melanoma tumor models which recapitulate many aspects of human immunology.^25^ However, human data will ultimately be needed to determine the applicability of these findings to human disease. Clinical trials to evaluate the immunologic impact of neoadjuvant RT + anti-PD-1/L1 in melanoma are planned. Additionally, given our studies were restricted to melanoma, other cancer types need to be investigated in the future to establish the generalizability of our findings. Finally, we do not fully interrogate the mechanism by which RT promotes stem-like T cell differentiation. This will be done in future studies.

## METHODS

### Mice

Six-week-old female C57BL/6 mice were purchased from the Jackson Laboratory. All mice were used in accordance with the Emory University Institutional Animal Care and Use Committee guidelines. Mice were sacrificed if they become sick, lethargic or had >10% weight loss prior to tumor volume defined endpoint. The B16F10 cell line was obtained from American Type Culture Collection (ATCC). A B16F10 cell line expressing the glycoprotein (GP) of the LCMV Armstrong strain was generated by lentiviral transduction as described previously.^21^ Briefly, the codon-optimized GP was cloned into the bicistronic replication deficient lentiviral vector pLVX- IRES-ZsGreen1 (Takara) followed by production of lentiviral particles in 293 T cells (ATCC) and lentiviral transduction of B16F10 cells. A stable B16F10GP cell line was established by sorting B16F10 cells expressing high levels of the green fluorescent protein ZsGreen1 using a FACS AriaII (BD Biosciences) 2 weeks after transduction. The cell line was grown in Dulbecco’s modified Eagle’s medium (DMEM) supplemented with 10% FBS, 100 U/ mL penicillin and 100 ug/mL streptomycin, 2 mM glutamine. The cells were cultured at 37°C with 5% CO_2_.

Tcf7^DTR-eGFP^ mice was created by CRISPR/Cas-mediated genome engineering (Taconic Biosciences). The gRNA to mouse Tcf7 gene, the donor vector containing “IRES-DTR-P2A-EGFP” cassette, and Cas9 mRNA were co-injected into fertilized mouse eggs to generate targeted knock-in offspring. F0 founder animals were identified by PCR followed by sequence analysis, which were bred to wildtype mice to test germline transmission and F1 animal generation. These mice were then bred to P14 mice to generate P14 Tcf7^DTR-eGFP^.

### Tumor irradiation

5×10^5^ B16F10GP cells were injected into the right flanks on day 0 and left flanks on day 3. After the tumor was palpable (10–12 days), the right tumors were irradiated using Small Animal Radiation Therapy (SmART+) system by Precision. During radiation, mice were anesthetized with an isoflurane-based anesthesia system. The radiation dose was 10 Gy × 1 fraction. The treatment protocol was planned by SmART ATP – Advanced Treatment Planning. Tumor sizes were assessed using calipers. Tumor volume was calculated according to the formula length × width × depth × 0.52. For FTY720 experiment, FTY720 was provided in the drinking water (2 μg/mL) 2 days prior to RT. FTY720 treatment was continued throughout the entire experimental course. For αPD-L1 treatment, it was administered i.p. at a dose of 200 μg per mouse. For T cell analysis, mice were sacrificed on Day 19 when tumor, spleen, blood, and TdLN were harvested.

### Adoptive T cell transfer

P14 cells were obtained from the spleen of P14 mice. B6 mice (CD45.1) underwent retro-orbital injection with 2.5X10^5^ P14 cells one day prior to B16F10GP tumor cell implantation. P14 DTR+/− were used for stem-like T cell depletion experiments. For the depletion of DTR expressing cells, Diphtheria Toxin (DT) was injected through i.p. 3 times at a dose of 50 mg/kg of body weight.

### Flow cytometry

Flow cytometric analysis was performed on a BD FACSymphony A3. Direct ex vivo staining and intracellular cytokine staining were performed as described previously with fluorochrome-conjugated antibodies.^21^ To detect tumor- specific CD8+ T cells, MHC- I tetramers were prepared as described previously.^21^ For intracellular detection of transcription factors such as T- cell factor-1 (Tcf-1), cells were surface stained for 30 min, fixed and permeabilized using the Foxp3 Fixation/Permeabilization Kit according to manufacturer’s instructions (eBioscience), followed by intracellular staining for 30 min. All staining was performed in a 96 well plate. Splenocytes were used for single color controls. FACS data were analyzed with FlowJo (V10.8) software.

### Cell sorting

For single-cell RNA sequencing, CD8+ PD-1+ CD44+ cells from the tumor-draining LNs were flow sorted on a FACSAria (BD) flow cytometer. Individual mice samples were hashed (BioLegend) and pooled for sequencing. For TCR sequencing, CD8+ PD-1+ CD44+ cells from tumors were flow sorted on a FACSAria (BD) flow cytometer. For stem-like P14s transfer, CD8+ PD-1+ CD44+ Tim3− cells from tumor-draining LNs were flow sorted on a FACSAria (BD) flow cytometer.

### Single-cell RNA sequencing

Single-cell RNA sequencing was performed by 10x Genomics Chromium Controller.

#### Pre-processing of single cell RNA-seq data:

The Cell Ranger Single Cell Software Suite (version 5.0.1) by 10x Genomics was used to perform de-multiplexing, barcode processing, and single-cell 3’ gene counting. Reads from each pool were then aligned to the mm10–2020 mouse genome (2020 release). The count data was processed and analyzed in *R* (version 4.2.1) as described below. To deconvolute the cells belonging to each sample we used the Seurat package (version 4.1.1) in R. The outputs derived from CellRanger were used to create two separate objects (one with the transcriptome alignment and one with the antibody plus hashtags (HTO) alignment). Initial objects were created using the function “Read10X”. We filtered both objects based on the cell barcode to keep only cells which were identified in both the transcriptome and in the antibody alignments. After this cell filtering, we used the function “CreateSeuratObject” to create a transcriptome-based Seurat object. The antibody derived data was filtered to maintain only the hashtag counts; later it was appended as a specific assay using the “CreateAssayObject” function. For cell demultiplexing we used the function “HTODemux” with default parameters in order to maximize the number of singlets detected. Individual single cells were finally filtered based on their assigned “HTO_classification.global”= “Singlet”. Antibody reads were then normalized using the Seurat function “NormalizeData” with the parameters “normalization.method” = “CLR” and “margin”=”2”, to indicate a normalization across cells.

#### Quality control of the scRNA-seq:

Low quality cells with a high percentage of mitochondrial gene counts (>~10%) and with <500 measured genes were excluded. To mitigate potential doublet inclusion, cells with UMI count above 40,000 and detected genes above 5,000 were removed. A total of 20 samples were sequenced and 38,578 single cells (Untreated, 9,239 cells; RT, 8,932 cells; anti-PD-L1, 10,502 cells; RT + anti-PD-L1, 9,905 cells) were kept for subsequent analyses. In addition, the Miller et al. and Huang et al. single-cell datasets were imported without modification as validation sets.^22,29^ After filtering, data in each cell was log normalized using Seurat’s ‘NormalizeData’ function (method = ‘LogNormalize’, scale.factor = 10,000), the 2,000 most variable genes were identified, and the ‘ScaleData’ function was used to scale and center the gene expression matrix after regressing out the heterogeneity associated with cell cycle and mitochondrial contamination. For each dataset, the number of principal components used for neighborhood graph construction and dimensional reduction was set at 20. Uniform Manifold Approximation and Projection (UMAP) visualization indicated cells from different samples were well mixed into the shared space.^38^

#### Annotation of cell clusters:

To simplify the analysis, prior annotations from published datasets were used as a reference to annotate cell clusters in our dataset.^22,29^ If prior annotations were not available, cellular identity was determined by finding differentially expressed genes for each cluster versus all other cells using the ‘FindAllMarkers’ function in Seurat (test.use= ‘wilcox’, min.pct=0.1, logfc.threshold=.5).

#### Building single-cell trajectory:

To construct cellular trajectories and infer pseudotime, we used Monocle 3, a tool optimized to infer trajectories of differentiation using single-cell expression data.^39^ Cells derived from the TdLN and with the highest expression level of *Tcf7 were chosen* as the ‘root’ of the trajectory. Monocle 3 ordered each cell along a learned trajectory according to its transcriptional progress. The default parameters in Monocle 3 were used for the analysis.

### TCR sequencing

Genomic DNA (gDNA) was extracted from sorted T cells using AllPrep DNA/RNA Micro Kit (QIAGEN) according to the manufacturer’s instructions. The isolated gDNA was sent to Adaptive Biotechnologies (Seattle, WA, USA) for TCR sequencing by immunoSEQ assays.

### Statistical analysis

All experiments were analyzed using Prism 9 (GraphPad Software). Summary graphs show means ± SEM. Statistical significance was determined by one way analysis of variance (ANOVA) or as detailed in the figure legend. P values of <0.05 were considered statistically significant (*).

## Data Availability

Source data are provided with this paper. All scRNA-seq data will be uploaded to publicly available database before publication. Other relevant data are available from the corresponding author upon reasonable request.
